# Phloretin supplementation ameliorates intestinal injury of broilers with necrotic enteritis by alleviating inflammation, enhancing antioxidant capacity, regulating intestinal microbiota, and producing plant secondary metabolites^☆^^☆☆^

**DOI:** 10.1016/j.psj.2025.105187

**Published:** 2025-04-29

**Authors:** Mengjun Wu, Meng Peng, Jiajia Zang, Shaochen Han, Peng Li, Shuangshuang Guo, Giuseppe Maiorano, Qunbing Hu, Yongqing Hou, Dan Yi

**Affiliations:** a Hubei Key Laboratory of Animal Nutrition and Feed Science, Wuhan Polytechnic University, Wuhan, 430023, China; b Engineering Research Center of Feed Protein Resources of Agricultural By-products, Ministry of Education, Wuhan Polytechnic University, Wuhan 430023, China; c Department of Agricultural, Environmental and Food Sciences, University of Molise, Campobasso, 86100, Italy; d Hubei Horwath Biotechnology Co., Ltd., Xianning 437099, China

**Keywords:** Phloretin, Broilers, Necrotic enteritis, Growth performance, Intestinal health

## Abstract

The present study aimed to explore the effects of dietary phloretin **(PT)** on growth performance, immune response, and intestinal function in broilers with necrotic enteritis **(NE)**. A total of 288 1-day-old Arbor Acres chicks were assigned to 3 groups, with 8 replicates per group and 12 chicks per replicate. Over 6 weeks, birds were fed a basal diet or the same diet supplemented with 200 mg/kg phloretin. Birds in the challenged groups were inoculated with coccildia during d 7 to 9 and *Clostridium perfringens***(CP)** during d 14 to 18. Results showed that CP and coccidia challenge reduced the average daily gain and average daily feed intake, increased the feed conversion ratio of broilers, induced inflammation and oxidative stress, and inhibited mRNA expression levels for genes associated with intestinal barrier and nutrient transporters (*P* < 0.05). PT addition to the feed improved growth performance at early phase improved intestinal morphology, and elevated antioxidant capacity via increasing the activity of total antioxidant capacity and superoxide dismutase in the ileum in broilers with necrotic enteritis (*P* < 0.01). Dietary PT regulated the intesetinal immune function as observed by the increases in the content of secretory IgA in the ileum and decreased cytokines (Interleukin-1β, Interleukin-10) (*P* < 0.05). Moreover, NE infection significantly disrupted the balance of intestinal flora, and led to a lower level of short-chain fatty acids such as butyric acid concentration in the ileum, while PT improved the microbiota structure, and increased the intestinal acetic acid and butyric acid concentration (*P* < 0.001). Furthermore, metabolomics analysis indicated PT treatment improve plant secondary metabolites contents like phloretin 2′-o-glucuronide. Additionally, we observed a significant positive correlation among PT, *Ligilactobacillus* and butyric acid, and a positive correlation between *Ligilactobacillus* and plant secondary metabolites. Overall, PT supplementation could improve growth performance and ameliorate intestinal injury in broilers with necrotic enteritis by enhancing the antioxidant capacity and immune function, regulating intestinal flora structure and producing plant secondary metabolites.

## Graphical abstract

**Figure:**
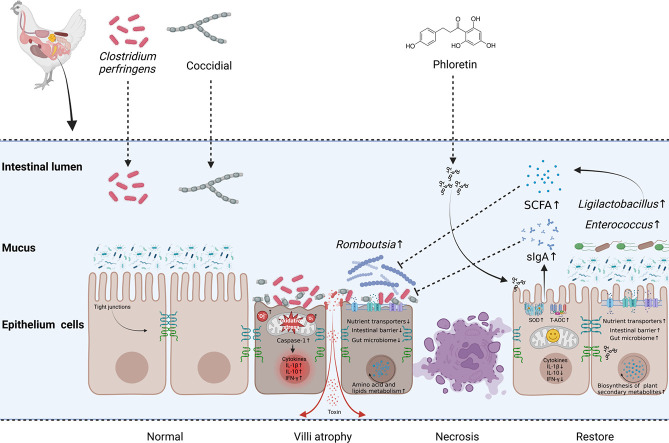

## Introduction

Necrotic enteritis (NE) is a significant intestinal disease in broilers caused by *Clostridium perfringens*, and it is often accompanied or occurs sequentially with coccidiosis during breeding (Abd El-Hack et al., 2022a; Caly et al., 2015). The NE could destroy the intestinal mucosa of chicks and cause dysfunction of nutrient absorption, significantly reduced growth performance (Chen et al., 2024; Xu et al., 2023a), and brought an estimated annual loss of approximately $6 billion worldwide (Emami and Dalloul, 2021; Fathima et al., 2022). Intestinal health problems of broiler chickens caused by NE hinder the high-quality and efficient development of intensive breeding and pose challenges to the sustainability of the poultry industry (Haque et al., 2020; Oviedo-Rondón, 2019). The use of antibiotic growth promoters was once an effective tool in protecting birds from enteric diseases. Nowadays, however, the removal of antibiotic growth promoters presents the poultry industry with several challenges and forces to look for potential alternatives to antibiotics in feed (Abd El-Hack et al., 2022b; Swaggerty et al., 2022).

Natural plant extracts have attracted more and more attention as functional feed additives for poultry nowadays. Elegant studies have shown that flavonoids have potential applications in livestock and poultry disease prevention and control, as well as improving intestinal health (Li et al., 2023; Pei et al., 2020). As an important flavonoid, phloretin (PT) has been found to possess a variety of biological activities including antioxidant, anti-inflammatory, bacteriostatic, and immunomodulatory function (Kumar et al., 2023). Specifically, PT was reported to improve antioxidant levels by regulating GSH-related enzymes, therefore improving the growth performance in heat-stressed broiler chickens (Hu et al., 2021). In recent years, the most important discovery of PT is that it could significantly inhibit intestinal inflammatory response (Habtemariam, 2023). The anti-inflammatory effect of PT in vivo has been confirmed in various enteritis models, including dextran sodium sulfate-induced standard mouse ulcerative colitis experimental model (Liu et al., 2021), acetic acid-induced rat ulcerative colitis experimental model (V.S. and S.K., 2021), and LPS-induced in vitro bovine rumen epithelial cell injury model (Wang et al., 2022). Intriguingly, PT plays an anti-ulcerative colitis role by restoring the integrity of the intestinal barrier, maintaining immune homeostasis, and improving intestinal flora disorders (Wu et al., 2019).

Nevertheless, PT may also have negative effects, such as affecting glucose metabolism by inhibiting glucose transporters (Habtemariam, 2023) and may cause liver damage (Itou da Silva et al., 2022). Among other things, whether adding PT to the diet can improve the growth performance and intestinal health of broilers with necrotic enteritis remains unclear. Therefore, the present study developed the broiler necrotic enteritis model co-stimulated by coccidia and *Clostridium perfringens* to explore whether phloretin exerts the beneficial effects on chicken growth and intestinal health. The results of the study would provide a theoretical basis and technical support for the practical application of phloretin in the broiler farming industry, which will contribute to the healthy and sustainable development of animal husbandry.

## Materials and methods

### Materials

Type A *C. perfringens* (CVCC2030) was obtained from the China Veterinary Microbial Culture Collection and Management Center (Beijing, China). The coccidial vaccine used in the present study was purchased from Foshan Zhengdian Biotechnology Co., Ltd. (Foshan, Guangdong, China), composed of 1 × 10^5^ oocysts of *E. acervuline* strain PAHY, and 5 × 10^4^ oocysts of *E. tenella* strain PTMZ, *E. maxima* strain PMHY, as well as *E. necatrix* strain PNHZ. Phloretin (≥ 98%, P834870) was provided by Shanghai Macklin Biochemical Technology Co., Ltd. (Shanghai, China).

### Experimental design, birds, and diets

The experiment was carried out at the poultry experiment base of Wuhan Polytechnic University (Wuhan, Hubei Province, China). A total of 288 1-day-old Arbor Acres chicks were weighed and randomly divided into 3 treatment groups based on similar body weight (average weight 41.675 g), half male and half female. There were eight replicate cages per treatment and 12 chicks per replicate. The chicks were allocated into: (i) negative control group (**CTR** group, basal diet); (ii) the coccidia and *C. perfringens* infected group (**CCP**, basal diet + coccidia and *C. perfringens* infection); (iii) Phloretin-treated and infected group (**CCP+PT**, basal diet plus 200 mg/kg phloretin + coccidia and *C. perfringens* infection). All chickens were vaccinated and managed (including light and temperature management) according to routine immunization and management programs of Arbor Acres broilers. Furthermore, all chicks were reared in wire cages and had free access to feed and water. The corn-soybean meal basal diets were formulated according to the recommendation of the Chinese chicken feeding standard (NY/T 33-2004) and were fed in the form of pellets, the formula of basal diet is summarized in Table S1. The trial was lasted for 43 days. The dosage of PT is chosen according to our preliminary study including 3 levels of PT (100, 200, and 400 mg/kg), which showed that 200 mg/kg PT increased the average daily gain of broilers from d1 to d21 of the age. All broilers were weighed at d 1, d 13, d 19 and d 43 of the age after a 12 h fasting. ADG, ADFI, and the feed conversion ratio **(FCR)** were calculated at different experimental periods. All animal procedures used in the present study were approved by the Institutional Animal Care and Use Committee of Wuhan Polytechnic University (Index number: WPU202206003). All animal experiments were conducted in compliance with the “Animal Research: Reporting of In Vivo Experiments” (ARRIVE) guidelines (https://arriveguidelines.org).

### Coccidia and C. perfringens Challenge

Necrotic enteritis was induced in chickens according to Guo et al. with slight modifications (Guo et al., 2023b). On d 7 and d 10 of the age, birds in the CCP and CCP+PT groups were orally administrated with attenuated coccidia vaccine, with 33,000 ± 3,300 spores (30-fold of recommended dose) per chick. Chicks in the CTR group received an equal volume of saline. Afterwards, chickens were orally gavaged with 1 mL *C. perfringens* type A CVCC2030 (1  ×  10^8^ CFU/mL) per day on d 14 to 18 of the age, except for broilers in the CTR group. The birds of CTR group received 1 mL sterile fluid medium.

### Sample collection

One chicken with the average body weight was selected from each replicate at d 13 and d 19 of the age, and blood was collected from the wing vein, euthanized by cervical dislocation, and then slaughtered for sample collection. The values for immune organ index were calculated by the following equation: (immune organ weight [g]/BW [kg]) (Choi et al., 2021). The blood was centrifuged at 3,000 × *g*, 4°C for 10 min to prepare serum, which was stored at -20°C for the assay of biochemical and immune parameters. The middle region (1 cm) of the jejunum and ileum was excised and immediately fixed in 4% paraformaldehyde for intestinal morphological examination (Guo et al., 2023a). Additionally, approximately 2 g of chyme from the middle ileum and colon was collected aseptically into sterile tubes, and the mucosa of the middle jejunum and ileum was then gently scraped with a sterile and cold slide, both of which were collected in sterile tubes and stored at -80°C until further analysis.

### Intestinal morphology

Intestinal histomorphology was assessed following the previous methodology (Yi et al., 2018). 1-cm-long small intestine samples were fixed in 4% paraformaldehyde. Then, the fixed samples were dehydrated and embedded in paraffin, sectioned at a thickness of 4 mm, and stained with haematoxylin and eosin. Morphological examination was conducted with a light microscope (Leica microsystems, Wetzlar, Germany) with the Leica Application Suite image analysis software (Leica microsystems, Wetzlar, Germany). Ten intact, well-oriented crypt-villus units were randomly selected and measured per section. Intestinal villus height (**VH**) and crypt depth (**CD**) were measured to calculate the ratio of villus height to crypt depth (**VH/CD**).

### Serum lysozyme and intestinal sIgA determination

The serum lysozyme activity (Cat. No. A050-1-1) was determined by using the commercial kit from Nanjing Jiancheng Bioengineering Research Institute Co., Ltd. (Nanjing, China). The contents of sIgA in the jejunum and ileum were determined by using Chicken sIgA ELISA kit (Cat. No. YM-s2632) purchased from Shanghai Yuanmu Bio-Technology Co., Ltd (Shanghai, China). Assays were performed in triplicate, under the instruction, according to the previous publication (Guo et al., 2023a).

### Antioxidant capacity and oxidation-relevant products in the serum and intestine

Serum, jejunum, and ileum samples were used for the analysis of anti-oxidative enzymes and related products. Activities of superoxide dismutase (**SOD**), total antioxidant capacity (**T-AOC**), catalase (**CAT**), as well as malondialdehyde (**MDA**) and hydrogen peroxide (**H_2_O_2_**) contents were determined by using commercially available kits (Nanjing Jiancheng Bioengineering Institute, Nanjing, China). Assays were performed in triplicate, under the instruction, according to the previous publication (Xu et al., 2024).

### RNA isolation and quantitative real-time PCR

The jejunal and ileal samples (100 mg) were placed in a 2-mL centrifuge tube, and RNA was extracted using RNAiso Plus (Takara, Dalian, China) reagent according to the manufacturer’s instructions (Li et al., 2024a). The purity of the extracted RNA was checked using a NanoDrop 2000 nucleic acid spectrophotometer (Thermo Fisher Scientific), and samples with OD260/OD280 greater than 1.8 were used for subsequent processing. Then cDNA was synthesized using PrimeScript RT reagent kit with gDNA Eraser (Takara, Dalian, China). The qRT-PCR was performed using the Tap Pro Universal SYBP qPCR Master Mix (Vazyme, Nanjing, China) on an Applied Biosystems 7500 Fast qRT-PCR System (Foster City, CA) under the following conditions: 95°C, 30s; 95°C, 5s; 60°C, 34s, a total of 40 cycles; 95°C, 15s; 60°C, 1min; 95°C, 15s. The glyceraldehyde-3-phosphate dehydrogenase (GAPDH) gene was employed as a reference in the present study, and the relative expression of the gene was calculated using the 2^-ΔΔCt^ technique reported previously (Yu et al., 2024). The primer sequences used for this study are listed in Table S2.

### Ileum microbiota

Genomic DNA was extracted by using a DNA extraction kit (Qiagen, Hilden, Germany). The purity and concentration of DNA were then tested using 1% agarose gel electrophoresis, and the sample was diluted to 1 ng/μL using sterile water and used as a template. The universal primers 515F and 806R of the 16S rDNA gene V4 region were used to identify bacterial diversity according to previously described methods (Song et al., 2022b). After amplification, PCR products run on a 2% agarose gel and were purified using a QIAquick Gel Extraction Kit (Qiagen, Germany). Purified amplicons were pooled in equimolar amounts, and their paired-end reads were sequenced on an Illumina HiSeq2500 PE250 platform (Illumina, San Diego, USA) at Novogene Bioinformatics Technology Co. Ltd. (Beijing, China). Sequence processing and bioinformatics analysis was conducted following a previous study (Song et al., 2022a). In brief, raw tags were generated by merging paired-end reads using FLASH software. High-quality clean tags were obtained by QIIME analysis, and chimera sequences were removed to obtain effective tags using the UCHIME algorithm. Sequences were analyzed by UPARSE software and clustered into operational taxonomic units (**OTUs**) at a similarity level of 97%. Each OTU was annotated with the Greengenes database. Beta diversity was evaluated by principal component analysis (**PCA**) to show the differences of bacterial community structures, and the significance of separation was tested via ANOSIM. PICRUSt analysis was used to predict the functional potential of bacteria communities. OTUs were normalized by copy number, and metagenome prediction was further categorized into Kyoto Encyclopedia of Genes and Genomes (**KEGG**).

### Untargeted HPLC/MS-based metabolomics

The metabolites were extracted from ileum content samples using a methanol: water (4:1, v/v) solution. A 2μL sample was separated by an HSS T3 column (100 mm × 2.1 mm i.d., 1.8 μm) before mass spectrometry detection. The mobile phases included 0.1% formic acid in water: acetonitrile (95:5, v/v) (solvent A) and 0.1% formic acid in acetonitrile: isopropanol: water (47.5:47.5:5, v/v) (solvent B). The conditions for the Thermo UHPLC-Q Exactive HF-X Mass Spectrometer were: heater temperature, 425°C; capillary temperature, 325 °C; sheath gas flow rate, 50 arb; aux gas flow rate, 13 arb; ion-spray voltage floating (ISVF), -3500V in negative mode and 3500V in positive mode; and normalized collision energy, 20-40-60V rolling for MS/MS. Full MS resolution was set at 60,000, and MS/MS resolution at 7500. Data acquisition was conducted in Data Dependent Acquisition (DDA) mode over a mass range of 70 to 1050 m/z. The raw LC/MS data were preprocessed with Progenesis QI (Waters Corporation, Milford, USA), and metabolites were identified by using the HMDB, Metlin, and Majorbio databases.

The raw data were then analyzed by using the Majorbio Cloud platform (cloud.majorbio.com) (Ren et al., 2022). After preprocessing, variance analysis was performed on the matrix file. PCA was conducted using the R package ropls (Version 1.6.2). Additionally, a student’s t-test and fold difference analysis were carried out. Significantly different metabolites were identified based on the Variable Importance in Projection score from the OPLS-DA model and the p-value from the student’s t-test, with criteria of VIP > 1 and *P* < 0.05. Differential metabolites between groups were mapped to their biochemical pathways by using KEGG database searches. Statistically significant pathways were identified by using Fisher’s exact test with scipy.stats (Python package).

### Short-chain fatty acids determination

Samples of cecal content (0.5 g) were thawed at room temperature and added with 1.5 mL of sterile demineralized water, then vortexed for 3 min. Subsequently, samples are centrifuged. 1 mL of supernatant was collected and added with 0.2 mL of 25% metaphosphoric acid solution into a new batch of Eppendorf, mixed thoroughly, then incubated at 4°C for 30 min. The supernatant was collected after centrifugation, filtered through 0.22 μm into a sample injection bottle, and then detected by Agilent 7980B gas chromatography analyzer (Agilent Technologies, Palo Alto, CA, USA) as described by a previous study (Garcia-Villalba et al., 2012).

### Concentrations of amino acids in blood

The levels of amino acids in the blood were determined as described by Xie et al. (Xie et al., 2013). In short, 1 mL of sample was thoroughly mixed with 1 mL of salicylsulphonic acid (2%) for 15 min on ice, and thereafter the supernatant was collected by centrifugation at 10,000 × *g* at 4°C for 15 min. The supernatant was mixed with Lithium hydroxide solution, and adjusted pH to 7.0, then filtered through a 0.22 µm filter membrane. The samples were further processed by the automated amino acid analyzer system (S433D, Sykam GmbH, Eresing, Germany) including an HPLC system (Waters Corporation, Milford, MA, USA). Mobile phases consisting of 0.1 mol/L sodium acetate (pH 7.2) and 100% methanol, respectively.

### Statistical analysis

Data were analyzed by one-way ANOVA by using SPSS 26.0 (SPSS, Inc., Chicago, IL, USA). Duncan’s test was applied to compare the differences among means. The results are shown as the mean ± standard deviation (SD). The *P* < 0.05 was considered statistically significant. The spearman's correlation coefficient was calculated to assess relationships between the metabolites and gut microbiota as well as SCFAs. Heatmaps were prepared to assess bivariate relationships between variables. *P < 0.05, **P < 0.01, ***P < 0.001.

## Results

### Growth performance

As shown in Table 1, from d1 to d13 of the age, coccidia chanllenge decreased the ADFI and ADG of broilers as compared to the CTR group (*P* < 0.05). However, dietary PT increased the ADFI and ADG in broilers challenged with coccidia treatment (*P* < 0.05). From d14 to d19 of the age, the ADFI and ADG were decreased, but FCR was increased in CCP group when compared with CTR group (*P* < 0.05). However, broilers in the CCP+PT group showed a lower ADFI and FCR compared to the CCP group (*P* < 0.05). From d20 to d43 of the age, the FCR decreased in the CCP group compared to the CTR group (*P* < 0.05), while FCR did not differ between CTR and CCP+PT. During the whole rearing period (from d1 to d43 of the age), CCP challenge reduced the ADFI and ADG of broilers (*P* < 0.05), whereas PT supplement inhibited the reduction of ADG in broilers challenged with CCP infection.

**Table 1:** Effect of phloretin on growth performance in CCP-challenged broilers.

Item	Diets^1^			
	CTR	CCP	CCP+PT	*P*-value
**d 1 to 13**				
ADFI, g/d	25.6±0.97^a^	22.73±0.34^b^	28.09±3.70^a^	0.003
ADG, g/d	20.92±0.53^a^	15.77±0.41^c^	18.52±1.49^b^	<0.001
FCR	1.25±0.02^b^	1.42±0.05^ab^	1.53±0.26^a^	0.018
**d 14 to 19**				
ADFI, g/d	69.00±1.56^a^	54.77±1.42^b^	48.38±7.12^c^	<0.001
ADG, g/d	52.43±2.09^a^	34.5±1.86^b^	38.18±4.99^b^	<0.001
FCR	1.34±0.03^b^	1.57±0.05^a^	1.20±0.21^b^	0.001
**d 20 to 43**				
ADFI, g/d	132.23±4.13	123.49±6.91	126.59±7.03	0.075
ADG, g/d	73.38±3.89	73.44±2.99	72.48±3.52	0.868
FCR	1.81±0.08^a^	1.72±0.03^b^	1.75±0.04^ab^	0.042
**d 1 to 43**				
ADFI, g/d	98.30±2.22^a^	88.86±4.52^b^	92.75±4.71^b^	0.003
ADG, g/d	53.95±2.06^a^	50.22±1.89^b^	51.2±3.04^ab^	0.042
FCR	1.82±0.08	1.8±0.03	1.81±0.03	0.743
**Final body weight, g**	2335.39±164.01	2197.74±113.37	2266.74±130.98	0.255

1 CTR, basal diet; CCP, basal diet + coccidia and *C. perfringens* challenge; CCP+PT, basal diet + 200 mg/kg phloretin + coccidia and *C. perfringens* challenge. These data are expressed as mean ± SD for a sample size of n = 8. ^a,b,c^Different letters superscripts in same row mean significant differences (*P* < 0.05). ADG = average daily gain; ADFI = average daily feed intake; FCR = feed conversion ratio.

### Intestinal morphology

**Fig. 1:**
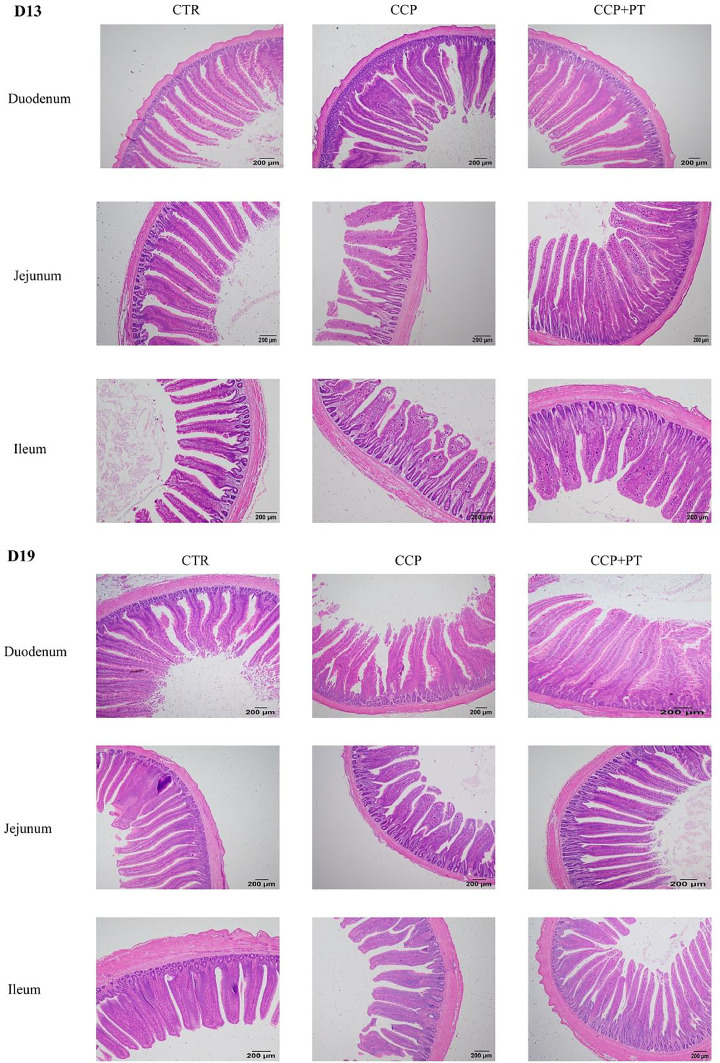
Effect of phloretin on intestinal morphology in CCP-challenged broilers. CTR, basal diet; CCP, basal diet + coccidia and *C. perfringens* challenge; CCP+PT, basal diet + 200 mg/kg phloretin + coccidia and *C. perfringens* challenge. The scale is 200 μm.

The microscopic pictures of intestinal mucosa are shown in Fig. 1. Tissue sections stained with H&E showed that the chickens in the infected group had intestinal histopathological changes with small intestinal villi damaged, lysed, as well as the intestinal epithelial cells were detached. The addition of PT to the diet improved the small intestinal morphology, indicated by increased intestinal villi compared with CCP group. The effects of phloretin on the intestinal morphology of broilers are shown in Table 2. On D13, compared with none-challenged broilers, CCP infection decreased the VH (duodenum and ileum), VH/CD (duodenum, jejunum and ileum), but increased CD in all small intestinal segments (*P* < 0.05). While PT addition increased CD (duodenum), VH and VH/CD (ileum), compared with the CCP group (*P* < 0.01). On D19, compared with none-challenged broilers, CCP infection decreased the VH/CD (duodenum), VH and VH/CD (jejunum), but increased VH (duodenum) and CD (duodenum and jejunum) (*P* < 0.01). Dietary PT increased VH and CD (duodenum), VH (jejunum) and CD (ileum), whereas decreased VH/CD (ileum) of CCP challenged broilers (*P* < 0.05).

**Table 2:** Effect of phloretin on intestinal morphology in CCP-challenged broilers.

Item	Diets^1^			
	CTR	CCP	CCP+PT	*P*-value
**d 13**				
Duodenum				
VH, μm	1351.69±114.84^a^	1161.34±98.45^b^	1243.73±122.72^ab^	0.010
CD, μm	136.67±17.49^c^	189.04±25.33^b^	248.35±24.54^a^	<0.001
VH/CD	10.33±0.68^a^	6.29±1.02^b^	5.36±1.00^b^	<0.001
Jejunum				
VH, μm	686.52±100.96	621.32±74.27	681.7±98.07	0.307
CD, μm	123.05±8.31^b^	199.71±31.41^a^	228.38±36.68^a^	<0.001
VH/CD	5.47±0.70^a^	3.31±0.80^b^	3.07±0.58^b^	<0.001
Ileum				
VH, μm	521.25±74.94^a^	410.93±72.86^b^	479.61±67.50^ab^	0.019
CD, μm	120.5±13.61^b^	198.11±24.59^a^	213.04±25.26^a^	<0.001
VH/CD	4.57±0.38^a^	2.08±0.51^c^	3.13±0.88^b^	<0.001
**d 19**				
Duodenum				
VH, μm	1145.35±62.93^c^	1311.05±117.42^b^	1470.88±89.20^a^	<0.001
CD, μm	120.29±6.90^c^	168.84±23.34^b^	233.09±21.20^a^	<0.001
VH/CD	9.62±0.84^a^	7.79±1.72^b^	6.59±1.01^b^	<0.001
Jejunum				
VH, μm	939.91±126.34^a^	757.74±65.69^b^	903.81±96.09^a^	0.003
CD, μm	152.03±18.76^b^	228.3±25.1^a^	245.49±49.67^a^	<0.001
VH/CD	5.74±0.59^a^	3.38±0.23^b^	3.84±0.71^b^	<0.001
Ileum				
VH, μm	643.51±78.88^b^	690.83±83.09^ab^	764.02±49.01^a^	0.011
CD, μm	149.24±20.66^b^	154.98±11.87^b^	194.32±26.27^a^	<0.001
VH/CD	4.89±0.41^a^	5.04±0.61^a^	4.24±0.59^b^	0.018

1 CTR, basal diet; CCP, basal diet + coccidia and *C. perfringens* challenge; CCP+PT, basal diet + 200 mg/kg phloretin + coccidia and *C. perfringens* challenge. These data are expressed as mean ± SD for a sample size of n = 8. ^a,b,c^Different letters superscripts in same row mean significant differences (*P* < 0.05). VH = villus height; CD = crypt depth.

### Organ index

The results of the organ index are shown in Table 3. On D13, compared with none-challenged broilers, coccidia challenge decreased the thymus gland index, but increased the liver index (*P* < 0.05). While compared with the CCP group, PT supplement increased the thymus gland and spleen index (*P* < 0.05) of broilers challenged with CCP. On D19, compared with none-challenged broilers, CCP infection increased the liver and spleen index (*P* < 0.01). While compared with the CCP group, diet PT decreased spleen index of CCP-infected broilers (*P* < 0.01).

**Table 3:** Effect of phloretin on organ index (g/kg) in CCP-challenged broilers.

Item	Diets^1^			
	CTR	CCP	CCP+PT	*P*-value
**d 13**				
Liver	29.78±1.12^b^	33.21±1.11^a^	33.69±1.70^a^	<0.001
Spleen	0.68±0.09^b^	0.73±0.16^b^	0.94±0.11^a^	0.001
Bursa of Fabricius	2.10±0.24	2.21±0.34	1.94±0.35	0.247
Thymus	1.69±0.13^a^	1.40±0.20^b^	1.59±0.20^a^	0.012
**d 19**				
Liver	26.04±0.93^b^	30.02±1.07^a^	29.10±0.68^a^	<0.001
Spleen	0.91±0.10^b^	1.20±0.15^a^	0.94±0.10^b^	<0.001
Bursa of Fabricius	2.41±0.49	2.49±0.31	2.71±0.26	0.243
Thymus	1.76±0.18	1.58±0.17	1.74±0.26	0.201

1 CTR, basal diet; CCP, basal diet + coccidia and *C. perfringens* challenge; CCP+PT, basal diet + 200 mg/kg phloretin + coccidia and *C. perfringens* challenge. These data are expressed as mean ± SD for a sample size of n = 8. ^a,b,c^Different letters superscripts in same row mean significant differences (*P* < 0.05).

### Intestinal sIgA and serum lysozyme

The results of the intestinal sIgA and serum lysozyme are shown in Table 4. Compared with none-challenged broilers, coccidia challenge increased the content of sIgA in the jejunum on D13, but decreased the contents of sIgA in the jejunum and ileum on D19. However, compared with the CCP group, CCP+PT increased the contents of sIgA in the ileum both on D13 and D19 (*P* < 0.05). In addition, Both CCP and CCP+PT groups increased the content of serum lysozyme on D13 and D19, compared with the CTR group (*P* < 0.01).

**Table 4:** Effect of phloretin on intestinal sIgA and serum lysozyme in CCP-challenged broilers.

Item	Diets^1^			
	CTR	CCP	CCP+PT	*P*-value
**d 13**				
Jejunal sIgA, μg/mgprot	2.70±0.51^b^	3.58±0.77^a^	3.55±0.76^a^	0.031
Ileal sIgA, μg/mgprot	4.07±0.89^ab^	3.45±0.57^b^	4.67±1.10^a^	0.039
Serum lysozyme, μg/mL	11.23±0.85^c^	12.66±0.61^b^	13.82±1.20^a^	<0.001
**d 19**				
Jejunal sIgA, μg/mgprot	7.26±1.56^a^	5.37±1.33^b^	5.42±0.56^b^	0.008
Ileal sIgA, μg/mgprot	5.78±0.75^a^	4.65±0.61^b^	5.40±0.62^a^	0.008
Serum lysozyme, μg/mL	13.33±1.41^b^	14.66±0.87^a^	15.49±1.08^a^	0.004

1 CTR, basal diet; CCP, basal diet + coccidia and *C. perfringens* challenge; CCP+PT, basal diet + 200 mg/kg phloretin + coccidia and *C. perfringens* challenge. These data are expressed as mean ± SD for a sample size of n = 8. ^a,b,c^Different letters superscripts in same row mean significant differences (*P* < 0.05).

### Intestinal antioxidant capacity

The results of antioxidant capacity in the jejunum and ileum are shown in Table 5. On D13, compared with none-challenged broilers, coccidia challenge increased the content of MDA in the ileum (*P* < 0.05). While compared with the CCP group, CCP+PT decreased MDA content, activity of CAT and SOD in the jejunum, as well as the activity of CAT in ileum (*P* < 0.05). On D19, compared with none-challenged broilers, CCP infection increased the content of MDA, the activity of T-AOC and SOD in jejunum, but decreased the activity of T-AOC and SOD in the ileum (*P* < 0.05). Compared with the CCP group, CCP+PT decreased the levels of MDA in the jejunum, but increased the activity of T-AOC and SOD in the ileum of broilers (*P* < 0.05).

**Table 5:** Effect of phloretin on intestinal antioxidant capacity in CCP-challenged broilers.

Item	Diets^1^			
	CTR	CCP	CCP+PT	*P*-value
**d 13**				
Jejunum				
MDA, nmol/mg prot	1.28±0.14^a^	1.35±0.14^a^	1.14±0.11^b^	0.011
CAT, U/mg prot	1.58±0.31^a^	1.53±0.23^a^	0.85±0.20^b^	<0.001
SOD, U/mg prot	564.43±35.08^a^	546.97±35.55^a^	465.61±34.40^b^	<0.001
Ileum				
MDA, nmol/mg prot	1.19±0.25^b^	2.67±0.63^a^	2.5±0.39^a^	<0.001
CAT, U/mg prot	16.5±2.08^a^	16.49±3.34^a^	2.93±0.64^b^	<0.001
SOD, U/mg prot	1026.07±128.23	917.32±135.01	918.98±152.53	0.224
**d 19**				
Jejunum				
MDA, nmol/mg prot	1.77±0.28^c^	3.38±0.66^a^	2.32±0.37^b^	<0.001
SOD, U/mg prot	298.82±50.27^a^	241.56±56.71^b^	199.02±21.21^b^	0.001
T-AOC, mmol/g prot	0.21±0.04^a^	0.14±0.02^b^	0.12±0.01^b^	<0.001
Ileum				
MDA, nmol/mg prot	1.24±0.36^b^	1.56±0.38^b^	2.49±0.49^a^	<0.001
SOD, U/mg prot	114.94±6.19^a^	98.06±6.00^b^	110.85±11.03^a^	0.001
T-AOC, mmol/g prot	0.23±0.01^a^	0.2±0.01^b^	0.23±0.02^a^	0.006

1 CTR, basal diet; CCP, basal diet + coccidia and *C. perfringens* challenge; CCP+PT, basal diet + 200 mg/kg phloretin + coccidia and *C. perfringens* challenge. These data are expressed as mean ± SD for a sample size of n = 8. ^a,b,c^Different letters superscripts in same row mean significant differences (*P* < 0.05). MDA = Malondialdehyde; CAT = Catalase; SOD = Superoxide dismutase; T-AOC = Total antioxidant capacity.

### Relative mRNA expression in the jejunum and ileum

**Fig. 2:**
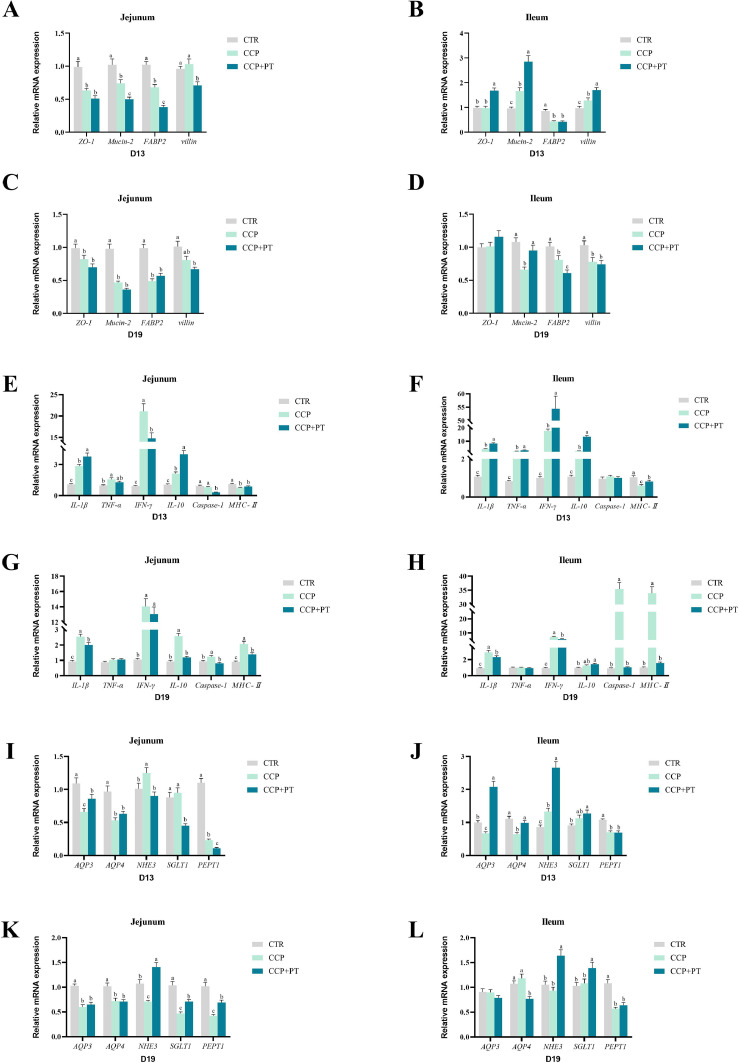
Effect of phloretin on relative mRNA expression of barrier-related genes in CCP-challenged broilers. A-D, barrier-related genes; E-H, immune-related genes; I-L, nutrient transport carriers genes. CTR, basal diet; CCP, basal diet + coccidia and *C. perfringens* challenge; CCP+PT, basal diet + 200 mg/kg phloretin + coccidia and *C. perfringens* challenge. These data are expressed as mean ± SD for a sample size of n = 8. ^a,b,c^Different letters superscripts mean significant differences (*P* < 0.05). ZO-1 = Zonula occludens-1; FABP2 = Fatty acid binding protein2; AQP3 = Aquaporin-3; AQP4 = Aquaporin-4; NHE3 = Na(+)-H(+) exchanger 3; SGLT1 = Sodium dependent glucose transporter1; PEPT1 = Oligopeptide transporter 1; IL-1β = Interleukin-1β; TNF-α = Tumor necrosis factor alpha; IFN-γ = Interferon gamma; IL-10 = Interleukin-10; MHC-Ⅱ = Major histocompatibility complex II.

The results of relative mRNA expression of barrier function-related genes in the jejunum and ileum are reported in Fig. 2 (A-D). On D13, compared with none-challenged broilers, coccidia challenge decreased the relative expression of *ZO-1, mucin-2*, and *FABP2* in jejunum; decreased the relative expression of *FABP2*, but increased the relative expression of *mucin-2* and *villin* in ileum (*P* < 0.01). However, compared with the CCP group, dietary PT increased the relative expression of *ZO-1, mucin-2*, and *villin* in ileum of broilers (*P* < 0.01). On D19, compared with none-challenged broilers, CCP infection decreased the relative expression of *ZO-1, mucin-2*, and *FABP2* in jejunum; decreased the relative expression of *mucin-2, FABP2*, and *villin* in ileum (*P* < 0.01). While compared with the CCP group, PT increased the relative expression of *mucin-2* in ileum of broilers (*P* < 0.01).

The results of relative mRNA expression of immune-related genes in the jejunum and ileum are shown in Fig. 2 (E-H). On D13, compared with none-challenged broilers, coccidia infection increased the relative expression of *IL-1β, TNF-α, IFN-γ*, and *IL-10* in the jejunum and ileum (*P* < 0.01). While compared with the CCP group, CCP+PT decreased the relative expression of *IFN-γ* and *Caspase-1* in jejunum (*P* < 0.01). On D19, compared with none-challenged broilers, CCP infection increased the relative expression of *IL-1β, IFN-γ, IL-10, Caspase-1*, and *MHC-Ⅱ* in the jejunum, as well as the relative expression of *IL-1β, IFN-γ, Caspase-1*, and *MHC-Ⅱ* in the ileum (*P* < 0.01). Compared with the CCP group, however, PT supplement decreased the relative expression of *IL-1β, IL-10, Caspase-1*, and *MHC-Ⅱ* in jejunum as well as the relative expression of *IL-1β, IFN-γ, Caspase-1*, and *MHC-Ⅱ* in the ileum of broilers (*P* < 0.01).

The results of relative mRNA expression of genes associated with nutrient transport carrier in the jejunum and ileum are shown in Fig. 2 (I-L). On D13, compared with none-challenged broilers, coccidia challenge decreased the relative expression of *AQP3, AQP4*, and *PEPT1*, but increased the mRNA level for *NHE3* in the jejunum and ileum (*P* < 0.01). However, dietary PT supplementation increased the relative expression of *AQP3* in jejunum, and the mRNA levels for *AQP3, AQP4*, and *NHE3* in the ileum (*P* < 0.01). On D19, compared with none-challenged broilers, CCP infection decreased the relative expression of *AQP3, AQP4, NHE3, SGLT1*, and *PEPT1* in jejunum, and the relative expression of *PEPT1* in the ileum (*P* < 0.01). While compared with the CCP group, broilers in the CCP+PT group exhibited the increased mRNA levels for relative expression of NHE3, SGLT1, and PEPT1 in jejunum, as well as the elevated NHE3 and SGLT1 mRNA levels in the ileum (*P* < 0.01).

### α-Diversity, β-diversity and abundance of ileal microbiological

**Fig. 3:**
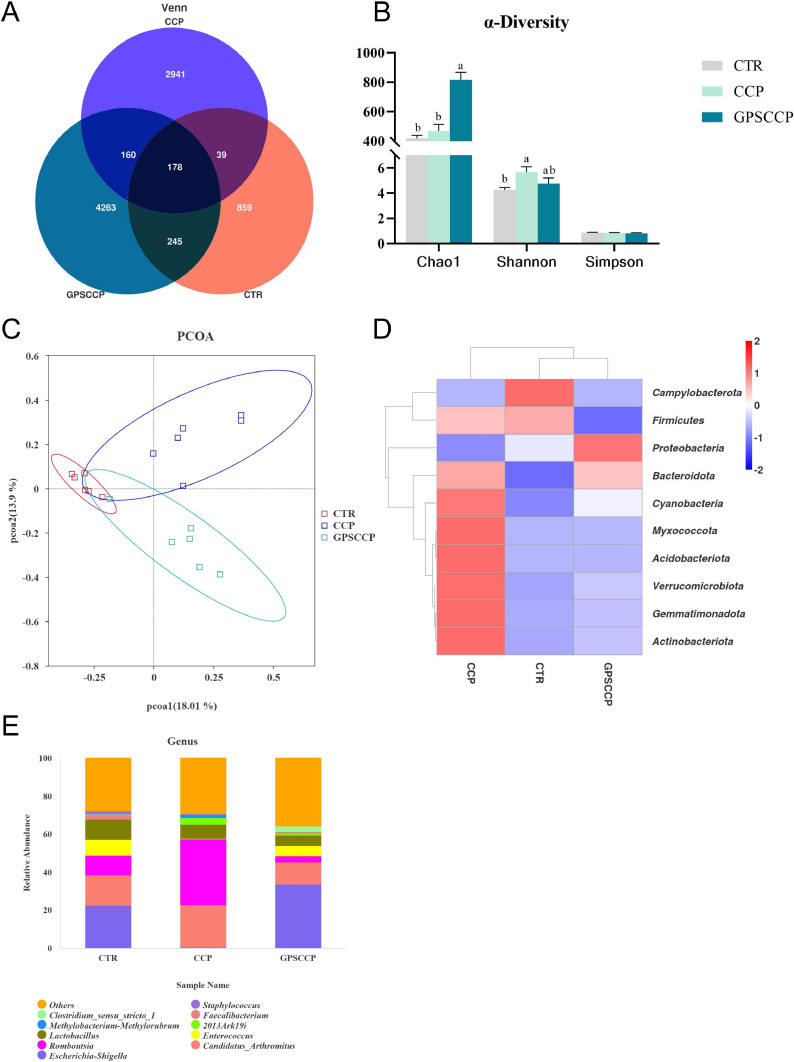
Effects of phloretin on the microbiota of the ileal midsection of broilers infected with CCP. A Venn diagram showing the OTUs of the ileal microorganisms. B α-diversity diagram showing ileal microorganisms. C, PCOA diagram. D and E, the differential species at phylum level and genus level. CTR, basal diet; CCP, basal diet + coccidia and *C. perfringens* challenge; GPSCCP, basal diet + 200 mg/kg phloretin + coccidia and *C. perfringens* challenge (n = 6 per experimental group). ^a,b,c^Different letters superscripts mean significant differences (*P* < 0.05).

To study the effect of phloretin on the ileal microbiota of NE broilers, the changes in the microorganisms were determined among the CTR, CCP, and GPSCCP (also known as CCP+PT) groups. There were 859 unique OTUs in the CTR group, 2941 in the CCP group, and 4263 in the GPSCCP group (Fig. 3A). Alpha diversity was measured using the Chao1, Simpson, and Shannon indices. Fig. 3B shows that CCP significantly increased the Shannon indices, compared with CTR group, while GPSCCP significantly increased the Chao1 indices, compared with CCP group. The principal component analysis (PCoA) of ileal microorganisms is shown in Fig. 3C. The principal component analysis showed there was a certain degree of dispersion between the 3 groups.

Fig. 3 also shown the effect of phloretin on the relative abundance of microorganisms in the ileum of NE broilers. At the phylum level, compared with the CTR group, CCP infection significantly increased the relative abundances of *Bacteroidota, Cyanobacteria, Myxococcota*, and *Acidobacteriota, Verrucomicrobiota, Gemmatimonadota,* and *Actinobacteriota*. However, compared with the CCP group, adding PT to the diet significantly reduced the abundances of *Cyanobacteria, Myxococcota, Acidobacteriota, Verrucomicrobiota, Gemmatimonadota* and *Actinobacteria* (Fig. 3D). The figure of PCoA combined with D showed that there were remarkable differences in the composition and structure of the ileal microbiota between treatment groups. In detail, Fig. 3E showed the top 11 species of genus horizontal abundance, indicated the difference between different microorganisms. At the genus level, CCP infection significantly reduced the relative abundances of *Escherichia-Shigella* and *Enterococcus*, but significantly increased the relative abundance of *Romboutsia*. However, adding PT to the diet significantly increased the relative abundances of *Escherichia-Shigella* and *Enterococcus*, while decreased the relative abundance of *Romboutsia* as compared with the CCP group.

### LEfSe analysis and functional prediction of ileal microbiota

**Fig. 4:**
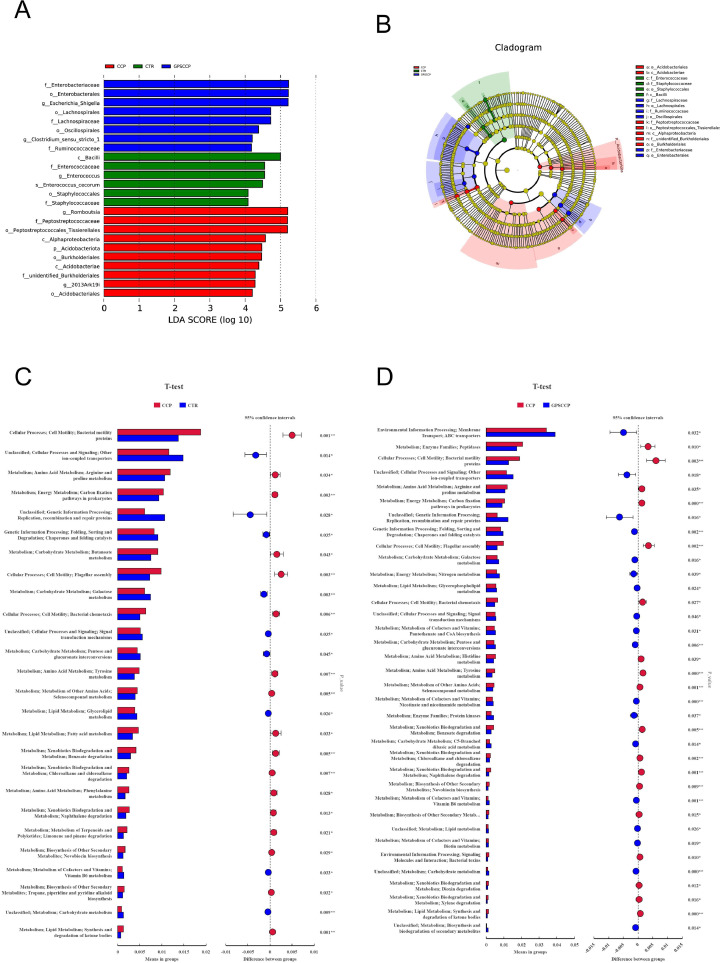
Effects of phloretin on linear discriminant analysis effect size and functional predictions of gut microbiota in CCP-challenged broilers. A, LDA analysis; B, cladogram analysis; C and D, Functional predictions. CTR, basal diet; CCP, basal diet + coccidia and *C. perfringens* challenge; GPSCCP, basal diet + 200 mg/kg phloretin + coccidia and *C. perfringens* challenge (n = 6 per experimental group).

Taxa that were significantly differentially represented among the groups were examined by LEfSe (LDA score = 4). Combined with the cladogram analysis results, in the CTR group, the dominant flora at the class level was *Bacilli*, the dominant flora at the order level was *Staphylococcales*, and the dominant flora at the family level were *Enterococcaaceae* and *Staphylococcacaae*. In the CCP group, however, the dominant flora at the class level were *Acidobacteriae* and *Alphaproteobacteria*, the dominant flora at the order level were *Acidobacteriales, Peptostreptococcales_Tissierellales*, and *Burkholderiales*, and the dominant flora at the family level were *Peptostreptococcaceae* and *unidentified_Burkholderiales*; the dominant flora at the genus level was *Romboutsia*. Interestingly, the dominant flora at the order level in the GPSCCP group were *Lachnospirales, Oscillospirales,* and *Enterobacterales*, and the dominant flora at the family level were *Lachnospiraceae* and *Enterobacteriaceae* (Fig. 4A-B).

In order to further explore the mechanism where phloretin exerts the beneficial effects on the intestinal function of broilers with NE, the present study conducted functional predictions based on changes in ileal flora (Fig. 4C-D). Compared with the control group, CCP infection significantly enhanced cellular processes, cell motility, metabolism, amino acid metabolism, arginine and proline metabolism, energy metabolism, carbon fixation pathways in prokaryotes, and flagellar assembly (*P* < 0.05), but weakened cellular processes and signaling, other ion-coupled transporters, genetic information processing, replication, recombination and repair proteins (*P* < 0.05). Compared with the CCP group, however, GPSCCP significantly heightened cellular processes and signaling, other ion-coupled transporters, genetic information processing, replication, recombination and repair proteins (*P* < 0.05), whereas dampened cellular processes, cell motility, metabolism, amino acid metabolism, arginine and proline metabolism, energy metabolism, carbon fixation pathways in prokaryotes, and flagellar assembly (*P* < 0.05).

### Untargeted LC/MS metabolomics

**Fig. 5:**
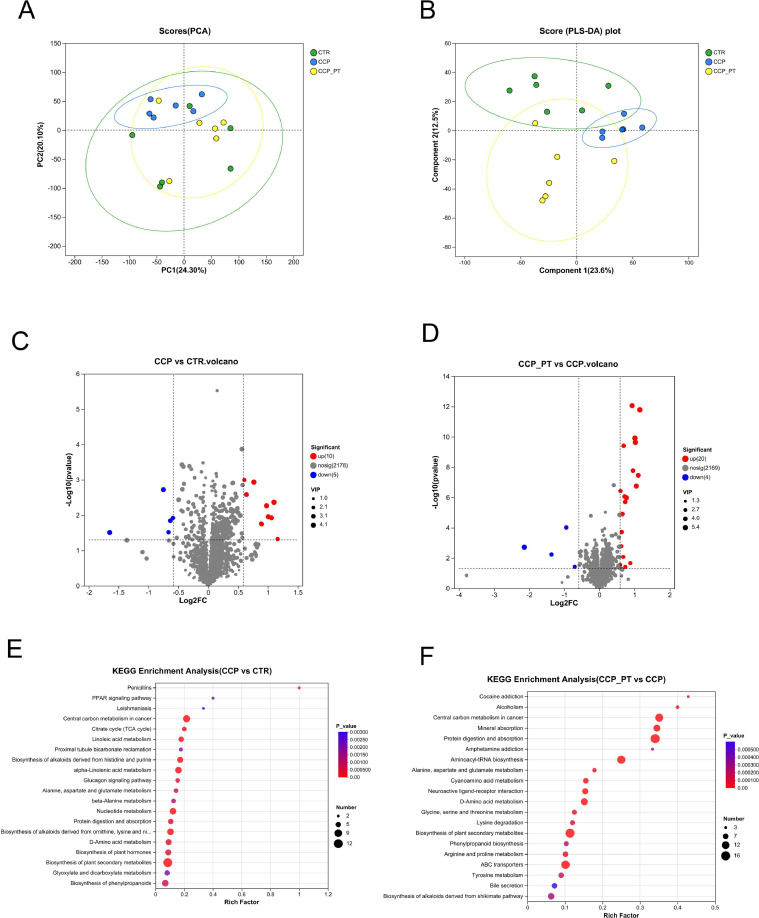
Effects of phloretin on ileal metabolome in CCP-challenged broilers. A, Principal component analysis (PCA) score plots; B, Projections to latent structure-discriminant analysis (PLS-DA) score plots; C and D, Volcano plots; E and F, KEGG enrichment analysis. CTR, basal diet; CCP, basal diet + coccidia and *C. perfringens* challenge; CCP_PT, basal diet + 200 mg/kg phloretin + coccidia and *C. perfrin* challenge (n = 6 per experimental group).

An untargeted HPLC/MS metabolomics analysis was conducted to analyze the differences in the ileum metabolites. PCA analysis showed metabolic variations among the 3 groups (PC1 = 24.3%, PC2 = 20.1%, Fig. 5A). Fig. 5B illustrated the PLS-DA analysis. Obvious changes were obtained: component 1 = 23.6%; Component 2 = 12.5%. Subsequently, the metabolites that contributed to the change in the metabolic composition among the groups were selected based on the strict thresholds of VIP score > 1, *P* < 0.05, and fold change >1.5 or < 0.67. As depicted in Fig. 5C-D, 15 differentially-abundant metabolites were identified from the comparison between the CTR and CCP groups (Table S3). 10 compounds were increased, and 5 compounds were decreased in the CCP group compared to those in CTR group. While 20 compounds were increased and 4 compounds were decreased in the CCP+PT group as compared to those in the CCP (Table S4). Further metabolic pathway enrichment analysis demonstrated that CCP significantly influenced the PPAR signaling pathway, Citrate cycle (TCA cycle), etc. While diet PT significantly altered the Central carbon metabolism, Mineral absorption, and Protein digestion and absorption (Fig. 5E-F).

### Levels of short chain fatty acids in the cecum chyme

On D19, CCP infection decreased the butyric acid levels in the cecal content of broilers as compared to the CTR group. However, PT supplement increased the levels of acetic acid and butyric acid in the cecum of broilers infected with CCP. The results were summarized in Table 6.

**Table 6:** Effect of phloretin on short chain fatty acids in the caecum of CCP-challenged broilers.

Item	Diets^1^			
	CTR	CCP	CCP+PT	*P*-value
Acetic acid, μg/g	1587.97±180.38^b^	1509.74±229.59^b^	2271.2±385.97^a^	<0.001
Propionic acid, μg/g	207.32±33.28	226.74±65.20	185.02±25.75	0.273
Isobutyric acid, μg/g	73.92±8.10	79.21±3.03	74.56±9.30	0.463
Butyric acid, μg/g	370.85±55.86^b^	244.32±40.78^c^	433.63±69.53^a^	<0.001
Isovaleric acid, μg/g	105.34±14.92	114.55±29.29	118.96±19.87	0.539
Valeric acid, μg/g	101.41±7.90	102.39±9.73	105.76±13.52	0.760

1 CTR, basal diet; CCP, basal diet + coccidia and *C. perfringens* challenge; CCP+PT, basal diet + 200 mg/kg phloretin + coccidia and *C. perfringens* challenge. These data are expressed as mean ± SD for a sample size of n = 8. ^a,b,c^Different letters superscripts in same row mean significant differences (*P* < 0.05).

### Concentrations of amino acids in blood

Data on free amino acid concentrations in blood was summarized in Table 7. CCP infection increased the concentrations of taurine, isoleucine, leucine, β-alanine, tryptophan, ammonia, and arginine in the blood of broilers. Dietary phloretin supplementation increased the contents of threonine, serine, glutamic acid, α-aminoadipic acid, α-aminobutyric acid, cystine, methionine, phenylalanine, β-aminoisobutyric acid, 3-methylhistidine, 1-methylhistidine, ornithine, lysine, ammonia and arginine, compared with the CCP group.

**Table 7:** Effect of phloretin on concentrations of blood amino acids in CCP-challenged broilers.

Contents, nmol/mL	Diets^1^			
	CTR	CCP	CCP+PT	*P*-value
*Essential amino acids*				
3-methylhistidine	31.79±5.8^b^	44.17±5.51^b^	145.6±26.68^a^	<0.001
1-methylhistidine	15.15±2.44^c^	23.29±2.64^b^	29.57±6.58^a^	<0.001
Isoleucine	92.87±10.88^ab^	108.78±23.25^a^	80.73±18.18^b^	0.028
Leucine	141.88±30.92^b^	178.85±37.45^a^	135.59±27.58^b^	0.037
Lysine	316.19±47.3^b^	369.71±68.6^b^	546.14±122.21^a^	<0.001
Methionine	55.47±5.08^b^	56.94±11.84^b^	79.55±16.89^a^	0.002
Phenylalanine	100.39±11.08^b^	111.03±10.89^b^	254.34±55.3^a^	<0.001
Threonine	207.09±33.25^b^	185.21±38.57^b^	270.93±67.43^a^	0.008
Tryptophan	8.75±1.95^b^	71.03±9.02^a^	79.38±11.6^a^	<0.001
Valine	149.89±17.85^b^	181.44±29.03^a^	143.23±29.52^b^	0.023
*Non-essential amino acids*				
Taurine	549.92±124.39^ab^	630.75±130.33^a^	427.09±91.66^b^	0.012
Phosphoserine	24.52±1.72	24.62±1.72	27.52±5.32	0.179
Serine	342.41±54.27^b^	321.29±76.1^b^	459.81±75.07^a^	0.003
Glutamic acid	163.98±12.85^b^	183.44±27.87^b^	325.73±51.87^a^	<0.001
α-aminoadipic acid	39.29±2.68^b^	40.75±7.3^b^	54.91±8.89^a^	0.001
Glycine	478.04±84.67^a^	506.12±115.73^a^	170.79±23.12^b^	<0.001
Alanine	1146.44±122.64^a^	1220.49±167.98^a^	682.77±152.33^b^	<0.001
α-aminobutyric acid	93.34±12.89^b^	101.22±11.93^b^	1470.79±321.71^a^	<0.001
Cystine	62.14±5.53^b^	72.32±12.43^b^	269.33±44.5^a^	<0.001
Tyrosine	205.85±31.31	188.48±31.06	188.87±41.72	0.569
β-Alanine	13.11±3.13^c^	35.42±7.98^b^	139.81±13.87^b^	<0.001
β-aminoisobutyric acid	27.13±6.19^b^	25.51±6.17^b^	41.79±6.32^a^	<0.001
Carnosine	20.56±4.48	26.05±5.73	23.89±4.96	0.142
Ornithine	40.92±6.89^b^	47.1±8.27^b^	69.63±14.79^a^	<0.001
Ammonia	660.93±19.95^b^	688.88±45.12^ab^	723.65±62.61^a^	0.067
Arginine	277.17±41.73^b^	335.73±77.53^ab^	386.08±73.49^a^	0.023

1 CTR, basal diet; CCP, basal diet + coccidia and *C. perfringens* challenge; CCP+PT, basal diet + 200 mg/kg phloretin + coccidia and *C. perfringens* challenge. These data are expressed as mean ± SD for a sample size of n = 8. ^a,b,c^Different letters superscripts in sam row mean significant differences (*P* < 0.05).

### Spearman correlation analyses

**Fig. 6:**
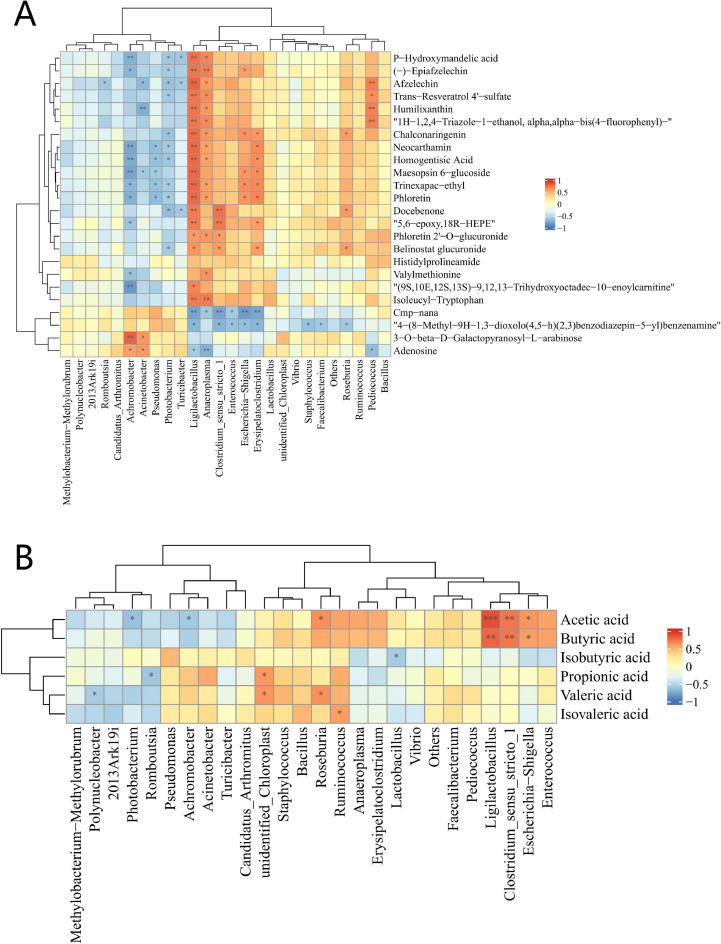
Heatmaps of Spearman's correlation analysis. A, Correlation of differential metabolites with growth and bacterial strains between CCP group and CCP_PT groups; B, Correlation of SCFAs with growth and bacterial strains between CCP group and CCP_PT groups. CCP, basal diet + coccidia and *C. perfringens* challenge; CCP_PT, basal diet + 200 mg/kg phloretin + coccidia and *C. perfringens* challenge (n = 6 per experimental group). *P< 0.05, **P< 0.01, ***P< 0.001.

Potential correlations among the metabolites, gut microbiota, and levels of SCFAs were investigated. A correlation analysis was conducted on factors with correlation coefficients (r) that were greater than 0.8 or less than -0.8. As shown in Fig. 6A, *Ligilactobacillus* showed a highly significant positive correlation with phloretin, Neocarthamin, Maesopsin 6-glucoside, Humilixanthin, and most of the metabolites (*P* < 0.01), and similar results were found also in *Anaeroplasma* (*P* < 0.05). On the contrary, *Achromobacter* showed a highly significant negative correlation with P-hydroxymandelic acid, Neocarthamin, Homogentisic Acid, etc (*P* < 0.01), similar results were found also in *Photobacterium* (*P* < 0.05). As for the correlations between the gut microbiota and levels of SCFAs, Fig. 6B suggested that acetic acid showed a highly significant positive correlation with *Ligilactobacillus* (*P* < 0.001) and *Clostridium_sensu_stricto_1* (*P* < 0.01), positive correlation with *Escherichia-Shigella, Roseburia* (*P* < 0.05), but negative correlation with *Photobacterium* and *Achromobacter* (P < 0.05). In addition, butyric acid showed a highly significant positive correlation with *Ligilactobacillus* (*P* < 0.001) and *Clostridium_sensu_stricto_1* (*P* < 0.01), and positive correlation with *Escherichia-Shigella* (*P* < 0.05).

## Discussion

Necrotic enteritis is characterized by the sudden onset of diarrhea and mucosal necrosis caused by the overgrowth of *C. perfringens* in the small intestine (Chen et al., 2024; Musa et al., 2019). NE was found to considerably reduce the ADG, ADFI and increase the FCR of broilers, and their small intestines showed remarkable pathological changes, such as intestinal congestion, red bruises, thinning of the intestinal wall, and intestinal distension. The intestinal section analysis further showed damage to the villi structure in the CCP group, which indicated the successful establishment of the necrotic enteritis (NE) model and was consistent with the results of previous studies (Xu et al., 2023a; Zhang et al., 2018). In addition, CCP impaired intestinal immune function and induced inflammation and oxidative stress, inhibited mRNA levels for intestinal barrier genes as well as nutrient transporters, and disrupted the balance of intestinal flora. PT addition to the feed had beneficial effects on the broilers with NE and improved intestinal morphology, and enhanced antioxidant capacity, immune function, as well as regulated intestinal flora.

### Effects of phloretin addition to the diet on the growth performance of broiler chickens with NE

Plant-derived bioactive compounds, as natural feed additives, have been used as alternatives to in-feed antimicrobials for years to improve the growth performance and health of broilers (Yang et al., 2017). There is some evidence suggesting that a inclusion of a flavonoid-rich feed additive, can partially help ameliorate the growth performance in NE broilers (Buiatte et al., 2022). Birds fed with PT had significantly improved ADFI and ADG and reduced FCR during the onset of NE (before d19 of the age), which is consistent with the results of previous studies. One study investigated the effects of dietary with 10-20 mg/kg grape seed extracts (GSPE) on *coccidia*-challenged broilers and found that significantly improved the growth performance, since GSPE addition into the poultry diet could decrease the concentration of plasma malondiadehyde and increased SOD activity, thus restoring the balance of the antioxidant/oxidant system that was exerted by the oxidative stress after the parasite infection (Wang et al., 2008). Researchers recently found 750 mg/kg tannic acid addition significantly increased the body weight on d19 and d28, but decreased the FCR of NE broilers throughout all trial periods . In addition, challenged birds fed diets supplemented with tannic acid showed significantly increased mRNA expression of nutrient transport carriers and intestinal barrier genes, indicating an enhancing intestinal barrier and absorption function (Xu et al., 2023a). Similar to our results, birds supplemented with a microencapsulated mixture of eugenol and garlic tincture showed better feed efficiency under a subclinical NE challenge (Kumar et al., 2022). However, during the finisher phase, the challenged birds (CCP group) seemed to recover somewhat from NE and the performance caught up with unchallenged birds (CTR group). Therefore, the finisher phase did not show PT effect on growth. This compensatory growth mechanism was also found in a previous study (Li et al., 2022b). In the present study, birds fed PT tended to improve ADG on d42, compared to CCP group. Similarly, the dietary supplement of ellagic acid (500 mg/kg) heightened ADG of broilers during d 22 to 42 and lowered FCR during d 22 to 42 and d 1 to 42 (Tang et al., 2022). However, the reasons that PT addition reduced the ADFI from d 14 to 19 are not clear, and maybe PT aggravated the negative effect of CCP on feed intake during this period. A previous study found that 100-200 mg/kg PT supplementation increased total feed intake in heat-stressed broilers (Hu et al., 2021). Similarly, 200 mg/kg PT supplementation increased total feed intake compared to the CCP group in the present study (3895.50g versus 3732.12g). The studies for investigating the interaction effect of diet PT and CCP treatment are warranted in further research. In addition, although the final BW was not significant, the dose of PT would be valuable in practice, considering the slaughter weight and following meat production (average final body weight: 2197.74 g in CCP group versus 2266.74 g in CCP+PT group). The presnt findings indicate that PT could ameliorate the reduction in growth performance of NE birds, possibly due to the improved intestinal health status. Previous studies found that PT may improve growth performance by enhancing antioxidant capacity through regulated GSH-related enzymes, Nrf2 and HSP70 in heat-stressed broilers (Hu et al., 2021). PT suppresses intestinal inflammation and maintained epithelial tight junction integrity by modulating cytokines secretion in an in vitro model of gut inflammation (Kapoor and Padwad, 2023). In addition, transcriptome analysis reveals the inhibitory mechanism of PT on virulence expression of Staphylococcus aureus, indicating a potential regulatory effect on intestinal microbiota (Li et al., 2024c). Therefore, the present study would further explore whether PT supplementation ameliorates intestinal injury of broilers with necrotic enteritis by alleviating inflammation, enhancing antioxidant capacity, and regulating intestinal microbiota.

### Effects of phloretin on the intestinal morphological structures, barrier function and nutrient transportation of broiler chickens with NE

Intestinal morphology is a key factor in maintaining intestinal health and integrity. An increased villus height may lead to enhanced nutrient absorption (Wang et al., 2021). In contrast, shorter villi and deeper crypts may result in lower disease resistance, poor growth performance, and malabsorption of nutrients (Mohammadagheri et al., 2016). Our findings suggested that CCP infection seriously destroyed the villi structure and reduced the absorption surface, also considerably increased crypt depth of intestine, which is consistent with previous findings (Du et al., 2016; Wu et al., 2018; Xue et al., 2018; Zhang et al., 2018). The addition of PT notably increased the villus height in the intestine of NE broilers on d 19 and improved the V/C ratio in ileum on d 13. Similar studies found that 500 to 1,000 mg/kg tannic acid was highly effective at ameliorating the abnormality in intestinal morphology (Xu et al., 2023a), it has been reported that thymol and carvacrol alleviated the ileal lesion via improving V/C ratio in broilers with *C. perfringens* infection (Du et al., 2016). In the mice model, the supplementation of phlorizin (a glucoside of phloretin) attenuated HFD-induced damage in epithelial integrity (Zhang et al., 2020). Therefore, the present study demonstrates that dietary PT could alleviate the abnormal intestinal histology of NE broilers.

The integrity of the intestinal barrier not only has a significant impact on the digestion and absorption of nutrients, but also plays a key role in protecting the intestine from infection of microorganisms (Du et al., 2016; Pirgozliev et al., 2019). Numerous studies found that the mRNA levels for tight junctions and nutrient transport carriers were down-regulated during intestinal inflammation, therefore impairing nutrient digestion and absorption (Gharib-Naseri et al., 2020; Kim et al., 2017; Pham et al., 2020; Zanu et al., 2020). In the present study, CCP infection resulted in a lower mRNA levels of barrier-related genes including *villin, ZO-1, MUC2*, and *FABP2*, causing an increase in intestinal permeability, in line with previous studies (Elshershaby et al., 2024; Graham et al., 2023; Tang et al., 2022; Xu et al., 2023a). Villin and FABP2 are primarily found in the microvilli of intestinal epithelial cells, implicated in forming and maintaining intestinal structures, the uptake and metabolism of nutrients (Xu et al., 2023a; Xu et al., 2023b). Tight junction proteins zonula occludens 1 play a crucial role in maintaining the integrity and function of epithelial cell barriers, including those found in the intestines of chickens (Graham et al., 2023). Mucin 2 is one of the major secreted mucins expressed by intestinal goblet cells and acts as a protective barrier for the intestine (Lee et al., 2018). In addition, CCP infection also decreased mRNA expression of nutrient transport carriers such as *AQP3, AQP4, NHE3, SGLT1, PEPT1*, suggesting the intestinal nutrient absorption may be weakened by CCP infection. Similarly, a previous study also observed a decreased mRNA expression of *PEPT1* and *SGLT1* in broiler chickens with NE (Guo et al., 2014). Notably, we first found *AQP3* and *AQP4* were also reduced in broilers infected with coccidia and *C. Perfringens*, indicating the detrimental influence of CCP infection on intestinal water transport or absorption in broilers. However, the mRNA expression of barrier-related and nutrient transport carriers genes, such as *NHE3* and *SGLT1* were up-regulated with the addition of PT compared to that in the CCP group, suggesting the positive effects of PT in improving the nutrient absorption in broilers.

### Effects of phloretin addition on the immune function on broiler chickens with NE

The organ indices of the liver, spleen, bursa of fabricius and thymus are important indexes to evaluate the immune function and health status of broilers (Choi et al., 2021; Yang et al., 2020). In the present study, CCP infection increased the liver and spleen indexes in broilers, which was consistent with previous studies that reported the enlarged liver and spleen of broilers can be induced by system inflammation in NE model (Shini et al., 2020; Song et al., 2022b; Xue et al., 2017). Intriguingly, we found that CCP infection could decrease the thymus index, while the addition of PT could increase the thymus index, and reach the same level as the CTR group. It is unclear whether necrotic enteritis causes immunosuppression of the spleen, but more studies reported the same results (Chen et al., 2023; Li et al., 2022b; Song et al., 2022b). A study found that phlorizin, a glucoside of PT, alleviated organ coefficient declines in aging mice (Chen et al., 2022). Similar to the present study, a previous found that 0.06% quercetin supplementation significantly increased the thymus index (Yang et al., 2020). Encapsulated thymol and carvacrol mixture, which are rich in flavonoids, also increased the thymus index of broilers (Li et al., 2022c). However, PT decreased the spleen index compared with the CCP group. The difference may be due to the infection procedure of coccidia and *C. Perfringens*, respectively. In addition, the increased serum lysozyme activity and reduced ileal sIgA contents in broilers indicated that CCP infection could cause systemic immune response in broilers, consistent with previous studies (Li et al., 2022b; Song et al., 2022b; Tang et al., 2022; Wang et al., 2017; Wang et al., 2023; Wang et al., 2021). Lysozyme can destroy cellular membrane integrity and result in cell death by cleaving peptidoglycan of the cell wall in Gram-positive bacteria (Wu et al., 2018). IgA protects the intestinal epithelium from bacteria, toxins, and viruses by neutralizing or preventing the binding of pathogens to the mucosal surface (Lammers et al., 2010). Furthermore, supplementation with PT increased the intestinal sIgA levels of NE broilers. Indeed, supplementation of polyphenols can enhance the level of intestinal sIgA and serum IgA levels (Huang et al., 2022). Specifically, quercetin was reported to dose-dependently increase the IgA content of Arbor Acre broilers (Yang et al., 2020). Therefore, PT supplement could heighten the immune function in broilers with NE.

Inflammation is the main immune response of the body; however, an excessive immune response may lead to an inflammatory cytokine storm, resulting in immune system dysfunction and therefore bringing irreversible damage to the host (Liu et al., 2020). *TNF-α* and *IL-1β* are pleiotropic pro-inflammatory cytokines, whose dysregulations are linked with a wide range of pathological conditions, such as infection, metabolic syndrome and inflammatory bowel disease (Kany et al., 2019). *IFN-γ* and *IL-10* also play an important role in a variety of inflammation-related diseases (Li et al., 2021; Zheng et al., 2020). Studies revealed that caspase-3-induced apoptosis (Wang et al., 2017), and the significant increase in the expression of *MHC-II* antigen-presenting molecules (Daneshmand et al., 2022) may also trigger inflammation responses in NE broilers. Here, we found that CCP infection increased mRNA levels for *IL-1β, TNF-α, IFN-γ, IL-10, caspase-3* and *MHC-II* in intestine, which were consistent with previous studies (Chen et al., 2024; Daneshmand et al., 2022; Wang et al., 2023), indicating severe intestinal inflammation responses in NE broilers. While the PT diet down-regulated the mRNA abundances of *TNF-α, IL-1β, IFN-γ, caspase-3* and *MHC-II*. A series of studies (Li et al., 2024b; Tang et al., 2022; Yang et al., 2020) have proved that phenols and flavonoids can relieve inflammation in the intestine of broilers. Meanwhile, the alleviating effects of phlorizin on inflammatory mediators have also been reported in mice, which is in line with our results and further indicates that PT reduced inflammatory responses in broilers probably through inhibiting the expression of inflammation cytokines. However, the addition of PT increased the expression of *IL-10* in this study, which may be because *IL-10* also serves as an anti-inflammatory cytokine, during the complex inflammation process (Chen et al., 2023). Anyhow, these results suggest that PT could regulate immune function and ameliorate inflammation responses of NE broilers.

### Effects of phloretin addition on the antioxidant capacity in broiler chickens with NE

Oxidative stress plays an important role in NE birds (Wang et al., 2017). Antioxidant enzymes like CAT, SOD, and T-AOC play a crucial role in scavenging free radicals in the body. The activity of these enzymes directly indicates the body’s antioxidant capacity and helps maintain the balance of free radicals (Cao et al., 2019; Kumar et al., 2012). In this study, CCP challenge decreased the antioxidant capacity of intestinal mucosa by reducing the activities of SOD and T-AOC, and increasing the concentration of MDA, which were consistent with previous studies (Tang et al., 2022). However, the dietary PT supplementation improved the activities of SOD and T-AOC, as well as decreased the contents of MDA in the jejunum. PT itself has shown a good antioxidant capacity in broilers, indicated by a lower serum MDA (Hu et al., 2021). Moreover, phlorizin could improve antioxidant enzyme activity while significantly reducing the MDA content, preventing of apoptosis in D-galactose-induced mice (Chen et al., 2022). Interestingly, PT reduced the activities of SOD and CAT in NE broilers on d13, even though there was no significant difference between CTR group and CCP group. The reason may be that intestinal oxidative stress was not obvious at the beginning phase during the NE challenge. Another two reports also demonstrated that the effects of NE challenge and supplementation with antioxidants in diet show different responses at different time points, sometimes converse (Li et al., 2022a; Li et al., 2024b). Similarly, an in vitro study found PT treatment decreased the mRNA level of CAT in human dermal fibroblasts, compared to the control group (Pawlowska-Goral et al., 2016). Nevertheless, considering the complexity of oxidation-redox reactions in the body, the present study indicated that dietary PT supplementation could improve the antioxidant capacity of NE birds. The antioxidant ability of PT is related to its effect on inducing changes in the redox state of cells (Han et al., 2020; Nithiya and Udayakumar, 2016).

### Effects of phloretin addition on the intestinal microflora in broiler chickens with NE

The intestinal microbiota forms a highly complex microecosystem. To further investigate the mechanisms by which PT alleviates the intestinal damage caused by CCP infection, we analyzed the microflora structure of the ileal microorganisms. The results of this study showed that the CCP challenge increased the Shannon index, which is in line with the results of previous studies (Chen et al., 2024; Wang et al., 2021). We speculated that CCP infection may increase the proliferation of microorganisms in the broiler intestinal flora. However, CCP challenge has also been shown not to disturb gut microbial α-diversity (Song et al., 2022b; Xu et al., 2023a), which may be related to the sample resource (e.g. jejunum, colon or cecum) and the time of sample collection. The significant difference in β-diversity among the 3 groups indicated that the PT diet or CCP infection significantly altered the microbial community structures. At the phylum level, CCP infection significantly increased the relative abundance of *Cyanobacteria, Myxococcota, Acidobacteriota, Verrucomicrobiota, Gemmatimonadota,* and *Actinobacteriota*. While addition of PT decreased the relative abundance of these microorganisms, reversed to a normal level similar to the CTR group. At the same time, PT addition significantly increased the relative abundance of *Proteobacteria*, which was decreased in the CCP group, consistent with previous studies (Yang et al., 2021). In addition, at the genus level, CCP infection significantly reduced the relative abundance of *Enterococcus*, but significantly increased the relative abundance of *Romboutsia*, similar to a previous study (Gautam et al., 2024; Zhao et al., 2022). Numerous studies have been performed to appraise the probiotic characteristics and potential of *enterococci*, suggesting its beneficial effect for intestinal health (Ferchichi et al., 2021). Adding PT to the diet significantly increased the relative abundance of *Enterococcus*, but decreased the relative abundance of *Romboutsia*. The latest research demonstrates that the increased genus *Rombutsia* may contribute to the inhibition of the proliferation of CP and progression of the infection to subclinical or clinical NE (Gautam et al., 2024). Considering the maintenance of the host-microbiota balance is the key to intestinal health and homeostasis in animals (Wu et al., 2020), the results indicate that the microbiota of the CTR group had a higher similarity with that of the PT group, but had a lower similarity with that of the CCP group, suggesting PT could restore the microflora of NE broilers to the CTR group. A similar study found that phlorizin improved the structure and diversity of the gut microbiota in mice (Chen et al., 2022). Furthermore, we observed a high significant positive correlation among PT, *Ligilactobacillus* and butyric acid. The systemic effects of the gut microbiota are attributed to the less studied SCFAs, which are produced in the gut as the final products of fiber fermentation and play an important role in the gastrointestinal tract of birds by inhibiting the growth of various pathogenic bacteria (Svihus et al., 2019; Wang et al., 2023). Researchers found that *Ligilactobacillus* strain produced acetic acid which was associated with inhibiting growth, adhesion, and invasion of *Salmonella* in a simulated chicken gut environment (Miri et al., 2023). Furthermore, this bacterium can re-establish the proper microbial balance of bacteria by forming lactic and propionic acid, stimulating butyric acid production, and inhibiting the production of pro-inflammatory cytokines (Yin et al., 2017). In the present study, dietary alleviated the inflammatory response in NE broilers, probably by increasing the abundance of *Ligilactobacillus* and stimulating the production of short-chain fatty acids, thus exerting anti-inflammatory effects and promoting intestinal health.

Based on the PICRUSt prediction of the difference in functional potential of the ileal microbiota, we found that differences in the metabolic pathways were the most common in this study, especially in the infection groups, when compared with the control groups. We speculated that metabolisms of amino acids, especially arginine and proline metabolism, may be enhanced in NE broilers. In agreement with our findings, the microbiota of chickens with severe NE had a stronger overall amino acid metabolism (Yang et al., 2021). Similarly, the finding was further validated in the present study by the determination of amino acid contents in blood. Given the fact that *C. perfringens* is predominant in intestine of NE chickens and lacks genes involving the biosynthesis of amino acids (Shimizu et al., 2002), thus amino acid synthetic pathways might be stimulated in the remaining bacterial population, to compensate and support the colonization of *C. perfringens* in the intestine of NE chickens. Moreover, the significant elevation of amino acids metabolism after CCP infection, suggests the property of *C. perfringens* to utilize amino acids from the host or other microbes in the intestine to support its growth. In fact, the increasing requirement for amino acids is also associated with enhanced NE susceptibility of chickens fed with a high protein diet (Moore, 2016). However, microbial catabolism of amino acids produces a large number of byproducts, including amines, phenols, and indoles (Abdallah et al., 2020), which may have detrimental effects on epithelial cells. On the other hand, PT addition significantly enhanced cellular processes and signaling, other ion-coupled transporters, genetic information processing, replication, recombination and repair proteins in ileal microbiota. In short, according to this study, the ileal flora structure and function of the PT groups were predicted to be similar to those of the CTR group chickens. Therefore, we conclude that the addition of PT could alleviate ileal microflora disorder in broiler chickens with NE.

### Effects of phloretin addition on the ileum metabolome in broiler chickens with NE

**Fig. 7:**
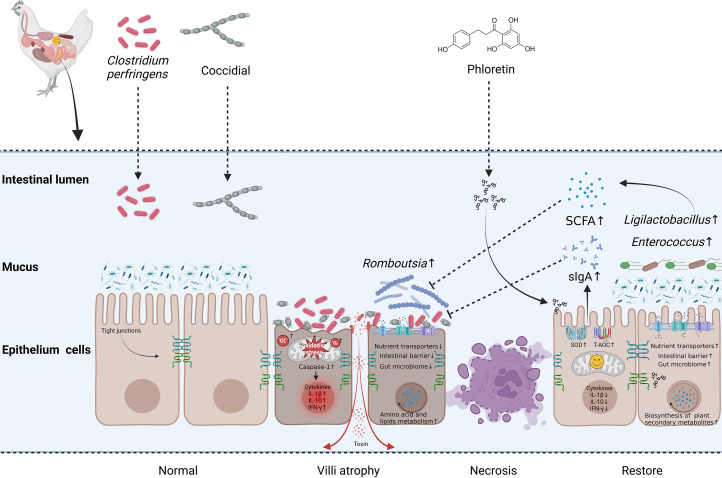
The schematic diagram of phloretin alleviating intestinal damage in broiler chickens with necrotic enteritis by enhancing intestinal barrier function and immune function, balancing microbial flora and improving metabolism (Created with Biorender.com).

More and more evidence shows that the release of metabolites from microbiota may influence the health of the host (Human Microbiome Project, 2012; Li et al., 2014). To further investigate that whether flora metabolites play the role in improving the intestine function by PT diet, we measured the nontarget metabolome of the ileal content. Our results revealed that 15 metabolites were significantly altered in NE broilers, including increased isocitric acid, 8-[(aminomethyl)sulfanyl]-6-sulfanyloctanoic acid, 4-methoxyestrone, anacardic acid, etc. These metabolites are not only involved in amino acid metabolic pathways, which were consistent with our finding of PICRUSt prediction analysis, but also participate in several important lipid metabolism such as alpha-linolenic acid metabolism, linoleic acid metabolism, as well as PPAR signaling pathway. Polyunsaturated fatty acids have long been associated with nutritional protection against intestinal inflammation (Bassaganya-Riera et al., 2002; Mills et al., 2005; Viladomiu et al., 2013). Previous study also found the modulation role of conjugated fatty acid on peroxisome proliferator-activated receptor gamma in obesity and inflammatory bowel disease (Yuan et al., 2015). The finding may indicate a potential protective mechanism of the broiler to alleviate the inflammation response during NE. In addition, pretreatment with PT significantly regulated the levels of 24 metabolites, including Homogentisic Acid, Neocarthamin, Phloretin 2′-O-glucuronide, Chalconaringenin, etc. Interestingly, the metabolites are primarily involved in the biosynthesis of plant secondary metabolites. Plant secondary metabolites, as anti-inflammatory agents, play an important role in protecting intestinal health (Russell and Duthie, 2011; Tammam et al., 2023). Phloretin 2′-O-glucuronide is a direct metabolite of phloretin, which has antioxidant and anti-inflammatory activities. Study found that bacteria-secreted homogentisic acid, could reduce inflammatory markers levels in LPS-induced RAW264.7 cells, showing potential applications in treatment for inflammation and cancer (Narayanan et al., 2019). Furthermore, correlation analysis revealed that *Ligilactobacillus* showed a highly significant positive correlation with homogentisic acid, neocarthamin, and chalconaringenin, and so did the *Anaeroplasma*, and *Erysipelatoclostridium*. The anti-inflammatory properties to induce the anti-inflammatory cytokine TGF-β, thereby also strengthening the intestinal barrier by enhancing mucosal IgA, qualify *Anaeroplasma* as a potent probiotic for the prevention and treatment of chronic inflammation (Beller et al., 2019). In short, consistent with the previous study, the present study has shown that phloretin can enter cells through active transport and paracellular absorption to exert its effects(Zhao et al., 2020). In addition, phloretin may also be metabolized by intestinal microorganisms into other phenolic acids (such as homogentisic acid) in the intestine, exerting anti-inflammatory and antioxidant effects, and helping to regulate intestinal health. Further studies are required to identify the biological impact of the plant secondary metabolites derived from phloretin. Whether or not, targeting these pathways and molecular confers a therapeutic effect against CCP infection, remains to be evaluated in future studies. To the best of our knowledge, this is the first study that suggests a positive correlation between *Ligilactobacillus* and plant secondary metabolites like homogentisic acid, neocarthamin, phloretin 2′-o-glucuronide, chalconaringenin, etc. in the NE broilers. The study would provide a theoretical basis for the treatment of NE with natural plant extract and also noted a potential value of *Rombutsia* and *Ligilactobacillus*, for fecal microbiota transplantation strategy. The schematic diagram of phloretin alleviating intestinal damage in broiler chickens with necrotic enteritis by enhancing intestinal barrier function and immune function, balancing microbial flora and improving metabolism was shown in Fig. 7.

## Conclusion

In summary, this study suggested that dietary phloretin alleviated CCP infection-induced intestinal injury in broilers via enhancing barrier function and immune function, balancing ileal microbiota and producing plant secondary metabolites, and finally enhancing the growth performance of broilers.

## Contributions

Dan Yi designed the study, Mengjun Wu and Meng Peng wrote the manuscript. Jiajia Zang, Shaochen Han, Peng Li, Qunbing Hu, and Shuangshuang Guo collected and analyzed experimental results. Giuseppe Maiorano, Binying Ding and Dan Yi participated in the revision of the paper. All authors contributed to the data interpretation and approved the final version of the manuscript.

## Supplementary data

Ileal chyme 16S rRNA data has been transmitted to the sequence read archive (PRJNA1182115). Ingredients and nutrient content of the chicken feed used during the trial (Table S1). Sequence of the oligonucleotide primers used for quantitative real-time PCR (Table S2). Effect of necrotic enteritis on the metabolome of broiler chickens (Table S3). Effect of phloretin supplementation on the metabolome of broiler chickens with necrotic enteritis (Table S4).

## Declaration of competing of interest

The authors declare that they have no known competing financial interests or personal relationships that could have appeared to influence the work reported in this paper.

## Appendix Supplementary materials

Image, application 1

## Footnotes

Supplementary material associated with this article can be found, in the online version, at doi:10.1016/j.psj.2025.105187.
