# Supplementary material for: Phloretin supplementation ameliorates intestinal injury of broilers with necrotic enteritis by alleviating inflammation, enhancing antioxidant capacity, regulating intestinal microbiota, and producing plant secondary metabolites
Source: Poult Sci. 2025 Apr 29;104(7):105187. doi: 10.1016/j.psj.2025.105187 (PMC12142525; doi:10.1016/j.psj.2025.105187)
Supplement: Supplementary file 1 [file mmc1.docx]

THE EFFECTS OF PHLORETIN ON BROILER CHICKEN

**Phloretin supplementation ameliorates intestinal injury of broilers with necrotic enteritis by alleviating inflammation, enhancing antioxidant capacity, regulating intestinal microbiota, and producing plant secondary metabolites**

Mengjun Wu^†,‡,*^, Meng Peng^#,*^, Jiajia Zang^†,‡,*^, Shaochen Han^†,‡^, Peng Li^†,‡^,

Shuangshuang Guo^†,‡^, Giuseppe Maiorano^#^, Qunbing Hu^||^, Yongqing Hou^†,‡^, Dan Yi^†,‡,1^

^†^Hubei Key Laboratory of Animal Nutrition and Feed Science, Wuhan Polytechnic University, Wuhan 430023, China;

^‡^Engineering Research Center of Feed Protein Resources of Agricultural By-products, Ministry of Education, Wuhan Polytechnic University, Wuhan 430023, China;

^#^Department of Agricultural, Environmental and Food Sciences, University of Molise, Campobasso 86100, Italy;

^||^Hubei Horwath Biotechnology Co., Ltd., Xianning 437099, China

^*^These authors share the first authorship

^1^Correspondence: [yidan810204@163.com](mailto:yidan810204@163.com)

The appropriate scientific sections for the paper are Immunology, Health and Disease; Metabolism and Nutrition

Table S1 Ingredients and nutrient content of the chicken feed used during the trial

|  | Starter (d 1-21) | Finisher (d 22-43) |
| --- | --- | --- |
| Ingredient, % |  |  |
| Corn (Grade 3 CP7) | 50.20 | 57.40 |
| Corn protein powder (Grade 3 CP<55) | 5.00 | 3.00 |
| Feather meal (hydrolyzed) | 1.00 | 1.00 |
| Soybean meal | 33.95 | 30.00 |
| Stone powder | 1.50 | 1.50 |
| Sodium chloride | 0.30 | 0.30 |
| Broilers-Mineral premix^1^ | 0.05 | 0.05 |
| Broilers-Vitamin premix^2^ | 0.05 | 0.05 |
| Lysine [98.5%] | 0.80 | 0.20 |
| DL-Methionine | 0.35 | 0.20 |
| Soybean oil | 5.00 | 3.00 |
| Calcium hydrogen phosphate | 1.50 | 1.50 |
| Choline chloride, 50% | 0.30 | 0.30 |
| Total | 100.00 | 100.00 |
| Nutrient content^3^ |  |  |
| Metabolic energy, mc/kg | 3.22 | 3.11 |
| Crude protein, % | 23.10 | 20.07 |
| Calcium, % | 1.11 | 1.10 |
| Available phosphorus, % | 0.45 | 0.45 |
| Lysine, % | 1.86 | 1.16 |
| Methionine, % | 0.68 | 0.49 |
| Methionine + Cystine, % | 1.03 | 0.81 |

^a^per kg of trace element premixed feed: copper, 8 g; iron, 80 g; zinc, 75 g; manganese, 100 g; iodine, 350 mg; selenium, 150 mg.

^b^per kg of vitamin premix feed: vitamin A12, 500 IU; vitamin D3, 2500 IU; vitamin E, 30 IU; vitamin K3, 2.65 mg; vitamin B1, 2.0 mg; vitamin B2, 6.0 mg; vitamin B12, 0.025 mg; biotin, 0.0325 mg; folic acid, 1.25 mg; niacin, 12 mg; nicotinic acid, 50 mg.

^c^Calculated values based on the experimental diet analysis

Table S2 Sequence of the oligonucleotide primers used for quantitative real-time PCR

| Gene | | Primer sequences(5ʹ→ 3ʹ) | NCBI serial number |  |
| --- | --- | --- | --- | --- |
| *GAPDH* | | F:GGTGAAAGTCGGAGTCAACGG | [NM_204305.2](https://www.ncbi.nlm.nih.gov/entrez/viewer.fcgi?db=nucleotide&id=2099396162) |  |
|  | | R:CGATGAAGGGATCATTGATGGC |  |  |
| *Intestinal barrier function-related genes* | | | |  |
| *ZO-1* | | F:CTTCAGGTGTTTCTCTTCCTCCTC | XM_040706827.2 |  |
|  | | R:CTGTGGTTTCATGGCTGGATC |  |  |
| *mucin-2* | | F:TTCATGATGCCTGCTCTTGTG | XM_040701656.2 |  |
|  | | R:CCTGAGCCTTGGTACATTCTTGT |  |  |
| *FABP2* | | F:GCAATGGGCGTGAATGTGA | [NM_001007923.2](https://www.ncbi.nlm.nih.gov/entrez/viewer.fcgi?db=nucleotide&id=2099367456) |  |
|  | | R:AGCCTGAAAGTTCAGTCCCGT |  |  |
| *villin* | | F:CAAGAGGCTACAGGAAGAGAATC | NM_205442.2 |  |
|  | | R:CCAAGTGTCCAGCAGGTAAA |  |  |
| *Nutrient transporter-related genes* | | | | |
| *AQP3* | | F:GCTTATCCTGGTGCTCTTCG | XM_046936218.1 |  |
|  | | R:GGCAAAGATACCAGCTGTGG |  |  |
| *AQP4* | | F:TGACTGCTTCTGCCTTGT | NM_001004765.3 |  |
|  | | R:AATGCTCCTGACTGTTCG |  |  |
| *NHE3* | | F:ATTGCCTCTTTTACCCTTAC | XM_040664559.2 |  |
|  | | R:CTGTCCAGGTGGCCCATTA |  |  |
| *SGLT1* | | F:TCAGGTCTACCTGTCAATCC | NM_001293240.2 |  |
|  | | R:GAGAATGAAAGATCCCACAA |  |  |
| *PEPT1* | | F:TACGCATACTGTCACCATCA | NM_204365.2 |  |
|  | | R:TCCTGAGAACGGACGTGAAT |  |  |
| *Immune and inflammation related genes* | | | | |
| *IL-1β* | F:ACTGGGCATCAAGGGCTA | | NM_204524.2 |  |
|  | R:GGTAGAAGATGAAGCGGG | |  |  |
| *TNF-α* | F:GAGCGTTGACTTGGCTGTC | | NM_204267.2 |  |
|  | R:AAGCAACAACCAGCTATGC | |  |  |
| *IFN-γ* | F:AGCTGACGGTGGACCTATT | | NM_205149.2 |  |
|  | R:GGCTTTGCGCTGGATTC | |  |  |
| *IL-10* | F:CGGGGAGCTGAGGGTGAA | | NM_001004414.4 |  |
|  | R:GTGAAGAAGCGGTGACAGC | |  |  |
| *Caspase-1* | F:CGGCCAGCGCCATCTTCAT | | AF031351.1 |  |
|  | R:AGGGAGCTGTCACAGTGCGT | |  |  |
| *MHC-Ⅱ* | F:ATAAGGCGTGGGCTCAGTTC | | [NM_001245061.1](https://www.ncbi.nlm.nih.gov/entrez/viewer.fcgi?db=nucleotide&id=350538722) |  |
|  | R:GAATTCGGGCAGCCTCCATA | |  |  |

GAPDH, glyceraldehyde-3-phosphate dehydrogenase; ZO-1, zonula occludens-1; FABP2, fatty acid binding protein2; AQP3, aquaporin-3; AQP4, aquaporin-4; NHE3, Na(+)-H(+) exchanger 3; SGLT1, sodium dependent glucose transporter1; PEPT1, oligopeptide transporter 1; IL-1β, interleukin-1β; TNF-α, tumor necrosis factor alpha; IFN-γ*,* interferon gamma; IL-10, interleukin-10; MHC-Ⅱ, major histocompatibility complex II.

Table S3 Effect of necrotic enteritis on the metabolome of broiler chickens

| Metabolite | *P*_value | VIP_PLS-DA | FC(CCP/CTR) | Regulate |
| --- | --- | --- | --- | --- |
| 8-[(Aminomethyl)sulfanyl]-6-sulfanyloctanoic acid | 0.004309 | 3.3787 | 2.1445 | up |
| LysoPC(14:1(9Z)/0:0) | 0.005435 | 3.129 | 1.9681 | up |
| PC(P-16:0/0:0) | 0.001162 | 3.1985 | 1.6994 | up |
| MALEIMIDE | 0.03096 | 2.8014 | 0.3186 | down |
| Anacardic acid | 0.01111 | 2.7734 | 2.008 | up |
| (9S,10E,12S,13S)-9,12,13-Trihydroxyoctadec-10-enoylcarnitine | 0.001902 | 2.8283 | 0.594 | down |
| 4-Methoxyestrone | 0.01194 | 2.7936 | 2.0851 | up |
| 1-Heptadecanoylglycerophosphoethanolamine | 0.01792 | 2.9033 | 1.8577 | up |
| 14-(Hydroxymethyl)-5,9-dimethyltetracyclo[11.2.1.0~1,10~.0~4,9~]hexadecan-5-ol | 0.002616 | 2.9999 | 1.5578 | up |
| Hexadecadienylcarnitine | 0.01435 | 2.4499 | 0.644 | down |
| 9-(5-Pentylfuran-2-yl)nonanoylcarnitine | 0.01734 | 2.5707 | 1.8496 | up |
| Glutathione episulfonium ion | 0.01199 | 2.234 | 0.6637 | down |
| Isocitric Acid | 0.04743 | 2.3382 | 2.2381 | up |
| Lithocholic acid glycine conjugate | 0.03039 | 2.1787 | 0.6304 | down |
| Furanone A | 0.001011 | 2.4074 | 1.5211 | up |

Table S4 Effect of phloretin supplementation on the metabolome of broiler chickens with necrotic enteritis

| Metabolite | *P*_value | VIP_PLS-DA | FC(CCP+PT/CCP) | Regulate |
| --- | --- | --- | --- | --- |
| Phloretin | 1.62E-12 | 4.978 | 2.2141 | up |
| Neocarthamin | 1.21E-10 | 5.3827 | 2.0107 | up |
| Cmp-nana | 0.001904 | 5.2486 | 0.227 | down |
| Maesopsin 6-glucoside | 2.29E-10 | 4.7509 | 2.029 | up |
| Humilixanthin | 1.76E-07 | 4.4415 | 2.0691 | up |
| (-)-Epiafzelechin | 8.70E-13 | 4.4146 | 1.9046 | up |
| Phloretin 2'-O-glucuronide | 1.05E-06 | 4.7581 | 1.7032 | up |
| Homogentisic Acid | 3.46E-08 | 4.2248 | 2.1494 | up |
| Afzelechin | 1.24E-05 | 4.3411 | 1.571 | up |
| 1H-1,2,4-Triazole-1-ethanol, alpha,alpha-bis(4-fluorophenyl)- | 8.68E-07 | 4.1307 | 1.6435 | up |
| P-Hydroxymandelic acid | 1.69E-08 | 3.7419 | 1.938 | up |
| Trans-Resveratrol 4'-sulfate | 1.97E-06 | 4.0932 | 1.6606 | up |
| Chalconaringenin | 3.85E-10 | 3.9607 | 1.61 | up |
| Adenosine | 9.60E-05 | 3.4288 | 0.5196 | down |
| Belinostat glucuronide | 0.0001913 | 3.8625 | 1.5509 | up |
| Trinexapac-ethyl | 3.75E-07 | 3.1961 | 1.5152 | up |
| 5,6-epoxy,18R-HEPE | 0.001664 | 3.1528 | 1.5411 | up |
| 4-(8-Methyl-9H-1,3-dioxolo(4,5-h)(2,3)benzodiazepin-5-yl)benzenamine | 0.005834 | 3.6763 | 0.3879 | down |
| (9S,10E,12S,13S)-9,12,13-Trihydroxyoctadec-10-enoylcarnitine | 0.008387 | 2.8711 | 1.5974 | up |
| Docebenone | 0.02159 | 2.9549 | 1.8364 | up |
| Isoleucyl-Tryptophan | 0.03873 | 2.9148 | 1.6647 | up |
| Valylmethionine | 0.02828 | 2.7109 | 1.5035 | up |
| 3-O-beta-D-Galactopyranosyl-L-arabinose | 0.03776 | 2.5624 | 0.6138 | down |
| Histidylprolineamide | 0.04943 | 2.9867 | 1.5408 | up |
